# Refinement and application of 12S *rRNA* meta-barcoding primers for seafood identification in multispecies product

**DOI:** 10.1016/j.crfs.2026.101486

**Published:** 2026-06-25

**Authors:** Feng Guan, Qingqing Hu, Siyu Yang, Shimeng Dai, Jian Ge, Xingjuan Hu

**Affiliations:** a College of Life Sciences, China Jiliang University, Hangzhou, Zhejiang, 310018, China; b Suzhou Novoprotein Scientific Inc, Suzhou, Jiangsu, 215299, China; c College of Education, Michigan State University, 620 Farm Ln, East Lansing, MI, 48824, USA; d Technology Center of Zhoushan Customs District, Zhoushan, Zhejiang, 316021, China

**Keywords:** Seafood, Fish ball, Ingredients identification, DNA meta-barcoding, 12S rRNA

## Abstract

Fish balls, processed from fish surimi, are a popular seafood product that has become a focus of food adulteration monitoring. To reveal the ingredients in fish balls and ensure authenticity, a set of meta-barcoding primers targeting the 12S rRNA gene was designed and optimized using mixed DNA samples from fish, livestock, and poultry, which are common ingredients in fish ball production. PCR products were sequenced using Next-Generation Sequencing (NGS) technology to identify animal-derived species. The results demonstrated that the primers accurately identified all mixed ingredients in various proportions. Nine batches of commercial fish balls were analyzed for species composition using the designed primers. All samples contained multiple fish species, with two batches showing serious discrepancies between actual ingredients and labeling. In conclusion, the optimized 12S rRNA meta-barcoding primers effectively identified the ingredients in multi-species fish balls, offering a complementary DNA barcoding tool for market regulation and consumer protection.

## Highlights

• High-throughput sequencing enables the accurate identification of fish balls ingredients.
• The 12S rRNA gene serves as an ideal meta-barcoding for fish species identification.
• The redesigned and optimized meta-barcoding primers targeting the 12S rRNA can detect all fish-derived ingredients in fish balls.
• High-throughput sequencing is incapable of quantitative detection of fish components in fish balls.

## Graphical abstract

**Figure:**
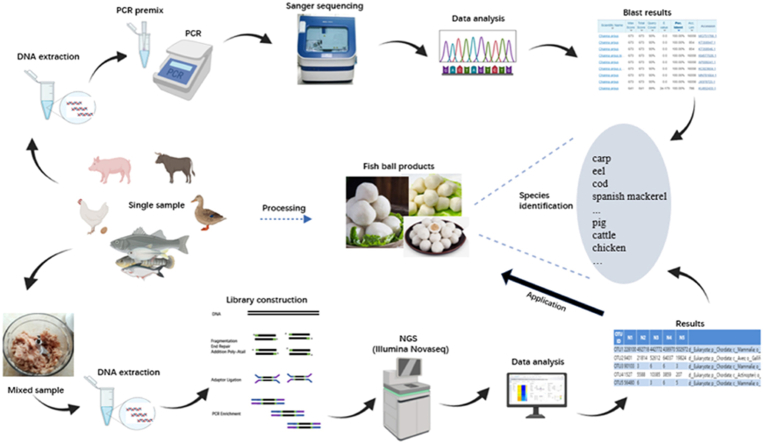

## 1 Introduction

Fish balls are ready-to-cook seafood products with rapidly growing sales, representing an alternative to whole fresh fish consumption. Fish balls are typically made from surimi mixed with starch, livestock and poultry meat, egg whites, and other condiments. These products are nutritious, convenient, and widely enjoyed in various regions of China and other Southeast Asian countries. A wide variety of fish, such as Alaska pollock, Atlantic cod, eel, horse mackerel, black carp, grass carp, silver carp, and bighead carp, can be used to produce fish balls (Huda and Ismail, 2009; Long et al., 2022).

Frozen surimi, an intermediate raw material for surimi products like fish balls, kamaboko, and chikuwa, is prepared through a series of processes, including filleting, deboning, washing, dehydrating, refining, blending with cryoprotectants, and freezing. Marine fish surimi has superior gel properties, resulting in higher-quality fish balls that are more expensive than those made from freshwater surimi (He et al., 2018). However, increasing surimi demand and decreasing marine fish availability have led some unscrupulous producers to adulterate products by mixing marine fish surimi with freshwater fish meat, adding excessive starch, non-muscle proteins, or hydrocolloids, or omitting fish ingredients entirely (Shumin et al., 2019; Giusti et al., 2017). Such practices violate food safety laws, harm consumers, and undermine market fairness. Due to the processed nature of fish balls, morphological identification of ingredients is impossible. Addressing this issue requires accurate and sensitive analytical methods for species identification.

DNA-based methods have been pivotal in identifying and differentiating species or cultivars in food, mainly targeting mitochondrial DNA (mtDNA). Techniques such as real-time PCR and DNA barcoding are favored for their high specificity, sensitivity, and reproducibility, making them suitable for identifying unknown species in food (Zhao et al., 2024; Rumiyati et al., 2024; Hu et al., 2025; Cichna-Markl and Mafra, 2023). However, these methods struggle to identify multiple unknown species in complex food matrices (Giusti et al., 2024; Zhang et al., 2024). Studies on animal-derived ingredients in mixed foods have shown that meta-barcoding combined with NGS can identify all target species in blended samples. This technology has become a key method for analyzing ingredients in complex processed foods, such as blended seafood (Zhang et al., 2024; Mottola et al., 2022; Piredda et al., 2022) and several animal products (Dobrovolny et al., 2019).

The mitochondrial 12S rRNA and 16S rRNA genes are widely used as meta-barcoding targets due to their conserved regions and high taxonomic resolution, making them effective for environmental DNA studies and lower organism identification (Zhong et al., 2022; Shu et al., 2021; Girish et al., 2022). Among these, the 12S rRNA gene is more suitable for amplifying fish DNA, as it has fewer primer-template mismatches and lower PCR amplification bias compared to 16S rRNA and Cytochrome Oxidase Subunit I (COI) genes (Girish et al., 2022; Jean, 2021; Zhang et al., 2020). Universal 12S rRNA primers offer similar taxonomic resolution as COI primers and are highly effective for high-throughput identification of common fish species (Palsson et al., 2022; Milan et al., 2020).

Given the multi-species composition of fish balls, DNA meta-barcoding is required for accurate ingredient identification. While 16S rRNA meta-barcoding has been used to identify species in processed foods like salmon products (Sarri et al., 2014; Wang et al., 2021), the capability of 12S rRNA meta-barcoding for this purpose remains unexplored.

This study aimed to design a novel set of 12S rRNA meta-barcoding primers for identifying fish, livestock, and poultry in fish balls. Three sets of universal primers targeting the 12S rRNA gene were developed and screened to identify the most suitable set, which was compared with 16S rRNA primers for analyzing fish ball ingredients. This approach established an NGS-based method for identifying unknown species in mixed and complex fish ball products.

## 2 Materials and methods

### 2.1 Sample collection

Based on published literature and label information, the composition of animal-derived ingredients in commercial fish balls from Hangzhou was preliminarily determined, providing data on fish species used in fish ball production. Additionally, fresh fish meat samples from 26 species (spanning 8 orders, 18 families, and 22 genera) and 4 other animal species were collected (Table 1). All samples were verified using morphological identification and DNA barcoding to confirm their species (Ivanova et al., 2007). Furthermore, 9 batches of commercial fish ball products were purchased from retailers and supermarkets to validate the developed assay.

**Table 1:** Information of collected fish and meat samples.

No.	Common name	Scientific name
1	grass carp	*Ctenopharyngodon idella*
2	silver carp	*Hypophthalmichthys molitrix*
3	bighead carp	*Hypophthalmichthys nobilis*
4	grouper	*Epinephelus awoara*
5	Black crucian carp	*Carassius auratus*
6	common carp	*Cyprinus carpio*
7	northern snakehead	*Channa argus*
8	largemouth bass	*Micropterus salmoides*
9	mandarin fish	*Siniperca chuatsi*
10	brown-marbled grouper	*Epinephelus fuscoguttatus*
11	Orange mouth anchovy	*Thryssa vitrirostris*
12	Japanese eel	*Anguilla japonica*
13	American eel	*Anguilla rostrata*
14	yellow catfish	*Tachysurus fulvidraco*
15	Pacific saury	*Cololabis saira*
16	*Pangasius bocourti*	*Pangasius bocourti*
17	large yellow croaker	*Larimichthys crocea,*
18	yellow croaker	*Larimichthys polyactis*
19	Large head hairtail	*Trichiurus japonicus*
20	silver pomfret	*Pampus argenteus*
21	Japanese spanish mackerel	*Scomberomorus niphonius*
22	chub mackerel	*Scomber japonicus*
23	bombay duck	*Harpadon nehereus*
24	elongate ilisha	*Ilisha elongata*
25	giant Grenadier	*Albatrossia pectoralis*
26	Atlantic cod	*Gadus morhua*
27	pig	*Sus scrofa*
28	cattle	*Bos taurus*
29	chicken	*Gallus gallus*
30	mallard	*Anas platyrhynchos*

### 2.2 Experimental design

#### 2.2.1 Preparation of artificial fish ball samples

Twelve fish species were selected for fish ball preparation based on common product label information. These species included silver carp, grass carp, argus snakehead, largemouth bass, grouper, mandarin fish, large yellow croaker, mackerel, Japanese eel, hairtail, Atlantic cod, and silver pomfret. The production process followed the standard fish ball manufacturing procedures (Jenkelunas and Li-Chan, 2018; Akter et al., 2013). Similarly, samples from four livestock and poultry species (pig, cattle, chicken, and duck) were processed for subsequent use. The basic processing procedure for fish ball production is as follows: Fish are dissected, rinsed thoroughly and dried. Muscle tissues behind the gills on the left side of the fish body are collected and cut into small cubes of approximately 1 cubic centimeter. Raw materials are blended in strict accordance with the weight ratio. Salt, egg white and corn starch are added at mass fractions of 0.2%, 2% and 1% relative to the total weight of fish muscle, respectively. The mixture is homogenized and comminuted with a meat grinder, then manually molded into fish balls. Subsequently, the fish balls are boiled in water for 15 min, cooled to room temperature, and set aside for subsequent use. These samples were uniformly mixed in varying proportions according to different mixing types. To evaluate the accuracy and sensitivity of DNA meta-barcoding, the mixing proportions for *S. niphonius*, *L. crocea*, and *C. idella* were set to a minimum of 1% each (Table 2).

**Table 2:** Preparation of manual fish ball samples.

No.	Number of mixed species	Species	Mixing proportions
N1	16	*C. Idella*, *H. molitrix*, *C. argus*, *M. salmoides*, *E. fuscoguttatus*, *S. chuatsi*, *L. crocea*, *S. niphonius*, *P. japonicus*, *T. japonicus*, *A. pectoralis*, *P. argenteus*, *S. scrofa*, *B. taurus*, *G. gallus*, *A. platyrhynchos*	Mix in equal proportion
N2	6	*S. niphonius*: *L. crocea*: *P. japonicus*: *C. Idella*: *S. scrofa*: *G. gallus*	34:1:20:5:20:20
N3	6	*S. niphonius*: *L. crocea*: *P. japonicus*: *C. Idella*: *S. scrofa*: *G. gallus*	20:5:34:1:20:20
N4	6	*S. niphonius*: *L. crocea*: *P. japonicus*: *C. Idella*: *S. scrofa*: *G. gallus*	10:20:10:20:20:20
N5	6	*S. niphonius*: *L. crocea*: *P. japonicus*: *C. Idella*: *S. scrofa*: *G. gallus*	1:30:0:30:20:19
N6	4	*S. niphonius*: *C. Idella*: *S. scrofa*: *G. gallus*	50:0:10:40
N7	4	*S. niphonius*: *C. Idella*: *S. scrofa*: *G. gallus*	40:10:40:10
N8	4	*S. niphonius*: *C. Idella*: *S. scrofa*: *G. gallus*	30:10:30:30
N9	4	*S. niphonius*: *C. Idella*: *S. scrofa*: *G. gallus*	0:40:10:50

#### 2.2.2 DNA extraction

Total genomic DNA from individual species was extracted using the Animal Tissue DNA Kit (Simgen Biotech Co., Ltd., Hangzhou). DNA from manually prepared and commercial fish balls was extracted using the Omega Tissue DNA Kit (Jinpan Biotech Co., Ltd., Shanghai) after pre-treatment with chloroform-methanol for oil removal (Shi et al., 2018). Extracted DNA samples were stored at −20°C.

All samples were processed in duplicate and analyzed using a NanoDrop 2000 (Thermo Fisher Scientific) to determine purity and concentration based on A_260_/A_280_ ratios. The higher-quality DNA samples were selected for further use.

#### 2.2.3 Primer design

The 12S rRNA sequences were analyzed using MegAlign software to identify conserved regions for primer design. Short mitochondrial 12S rRNA fragments (∼100–400 bp) were selected for DNA barcoding, as the read length of the Illumina Novaseq sequencing platform is limited to <400 bp. Reference sequences for the 12S rRNA of 20 related animal species were downloaded from the National Center for Biotechnology Information (NCBI) database, including *Larimichthys crocea* (NC_011710.1), *Larimichthys polyactis* (NC_013754.1), *Trichiurus haumela* (NC_042168.1), *Gadus morhua* (NC_002081.1), *Anguilla anguilla* (NC_006531.1), *Ctenopharyngodon idella* (NC_010288.1), *Hypophthalmichthys molitrix* (NC_010156.1), *Hypophthalmichthys nobilis* (NC_010194.1), *Carassius auratus* (NC_006291.1), *Cyprinus carpio* (NC_001606.1), *Engraulis japonicus* (NC_003097.1), *Siniperca chuatsi* (NC_015822.1), *Ilisha elongata* (NC_009585.1), *Scomber japonicus* (NC_013723.1), *Micropterus salmoides* (NC_008106.1), *Sus scrofa* (NC_000845.1), *Bos taurus* (NC_006853.1), *Ovis aries* (NC_001941.1), *Gallus gallus* (NC_053523.1), and *Anas platyrhynchos* (NC_009684.1).

**Fig. 1:**
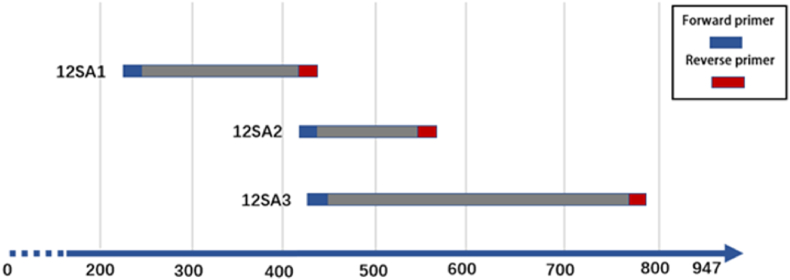
Diagram for primers location on mitochondrial *12S rRNA* gene (referenced GenBank NC 011710.1).

Three sets of universal primers (named 12SA 1–3) were designed using Primer 5.0 software and tested for specificity and universality using BLAST (https://blast.ncbi.nlm.nih.gov/Blast.cgi) (Fig. 1). Additionally, 16S rRNA universal primers were used as a reference to evaluate the accuracy of species identification and for comparative analysis (Sarri et al., 2014). All primers were synthesized by Tsingke Biotechnology Co., Ltd. (Hangzhou, Zhejiang) and diluted to 10 μM for use. Primer details are shown in Table 3.

**Table 3:** Information of primers used in this study.

Target genes	Primer name	Primer sequences (5’∼3′)	size (bp)	Tm(°C)
12S rRNA	12SA1	For-GGTAAAWCTCGTGCCAGCCA	207	60
		Rev- GGGGTATCTAATCCCAGTTT		
	12SA2	For-ACTGGGATTAGATACCCC	145	58
		Rev-TTMTAGAACAGGCTCCTCTA		
	12SA3	For-GGATTAGATACCCCACTATG	363	61
		Rev-TTACTRCTAAATCCWCCTT		
16S rRNA	16S rRNA	For-AYAAGACGAGAAGACCC	249	53
		Rev-GATTGCGCTGTTATTCC		

#### 2.2.4 PCR amplification and primers selection for single species

PCR amplification for each sample was performed using the primers listed in Table 3. Reactions were carried out in a PCR thermal cycler (Applied Biosystems, USA) with a total volume of 20 μL, containing 2 μL of 10× buffer (MgCl_2_-free), 2 μL of MgCl_2_ (25 mM), 1.6 μL of mixed dNTPs (2.5 mM), 1.0 μL of each primer (10 μM), 0.4 μL of *Taq* DNA Polymerase (5 U/μL), 4 μL of DNA template, and H_2_O to reach 20 μL. The PCR conditions were as follows: initial denaturation at 95°C for 5 min; 32 cycles of denaturation at 95°C for 40 s, annealing at the corresponding temperature (Table 3) for 40 s, and extension at 72°C for 1 min; final extension at 72°C for 10 min; and a hold at 4°C.

The PCR products were evaluated under ultraviolet light after electrophoresis in a 1% agarose gel stained with GelRed. Positive PCR products from single species were purified using a PCR purification kit. Sequencing of the purified products in both directions was conducted with the BigDye™ Terminator v3.1 Cycle Sequencing Kit (Thermo Fisher) on an ABI 3500 XL Sequencer. Sequencing was performed at Tsingke Biotechnology Co., Ltd. (Hangzhou, Zhejiang Province, China). The most suitable primers were selected for amplifying fish ball DNA based on universality, specificity, and species identification ability.

#### 2.2.5 PCR amplification and NGS for fish balls

After comparing the amplification and identification results from the three sets of primers, the 12SA3 primer set was selected for amplifying DNA from both manually prepared and commercial fish ball samples. Amplifications were performed on a GeneAmp® 9700 PCR System (ABI) in a total volume of 20 μL per reaction, containing 4 μL of 5× FastPfu Buffer (Mg^2+^ Plus) (TransGen Biotech Ltd.), 2 μL of 2.5 mM dNTPs, 0.8 μL each of forward and reverse primers (10 μM), 0.4 μL of FastPfu Polymerase (2.5 U/μL; TransGen Biotech Ltd.), 3 μL of DNA template (10∼20 ng), and H_2_O to 20 μL.

PCR conditions included initial denaturation at 95°C for 5 min; 32 cycles of denaturation at 95°C for 30 s, annealing at 55°C for 30 s, and extension at 72°C for 45 s; followed by a final extension at 72°C for 45 s and a hold at 4°C. Triplicate PCRs were performed for each sample and mixed post-amplification to reduce potential PCR bias. PCR products were detected on a 2% agarose gel.

**Fig. 2:**
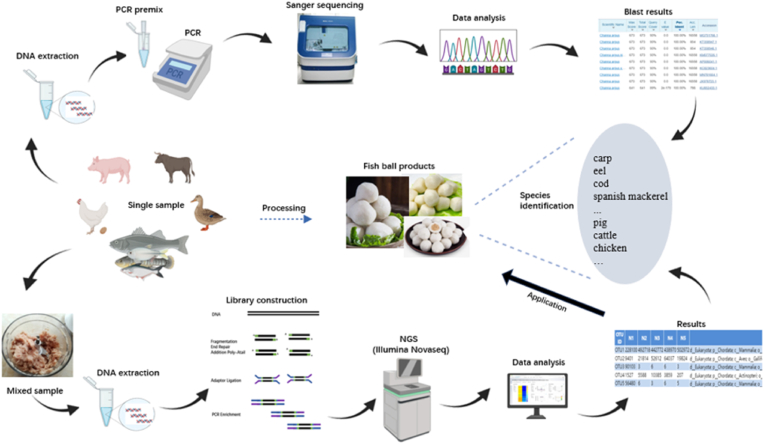
Experimental process related to meta-barcoding: Sanger sequencing and next generation sequencing.

Positive PCR products were purified using the AxyPrep DNA Gel Extraction Kit (Axygen Biosciences), quantified with a QuantiFluor™-ST (Promega), and mixed at equimolar concentrations. Library construction was performed using the Illumina library builder kit (Vazyme). Sequencing was carried out on the Illumina Novaseq 6000 SP (PE250) platform at Tsingke Biotechnology Co., Ltd. (Hangzhou) (Ma et al., 2022). The PCR and sequencing procedure is illustrated in Fig. 2.

#### 2.2.6 Statistical analysis

DNAStar software was used to splice and align the obtained sequences, which were then analyzed using BLAST on GenBank (http://blast.ncbi.nlm.nih.gov/Blast.cgi), applying a threshold of 98% for species identification (Pinto et al., 2015).

The NGS results underwent quality control, filtering, and splicing to obtain valid sequences. These sequences were clustered into operational taxonomic units (OTUs) at 97% identity using Usearch v.10 (Torbjørn et al., 2016) (http://drive5.com/uparse/). Taxonomic groups were assigned to OTUs with ≥98% identity and an E-value of ≤1 × 10^−1^ using the Blastn tool.

Based on the uclust algorithm, high-frequency sequences within each OTU were selected as representative sequences for species identification (Bhat et al., 2019). Sequencing reads for species in each sample that did not meet the specified criteria were labeled as unclassified. The software analysis was performed following standard analytical procedures. For NGS sequencing, a preliminary experiment is required to optimize the sequencing reaction conditions before starting the formal experiment. And each fish ball sample was sampled and sequenced at least twice. Species identification analysis was only performed when consistent results were obtained from the two replicates. Otherwise, a third round or further replicate tests were carried out.

## 3 Results and analysis

### 3.1 DNA barcoding analyses of single sample

#### 3.1.1 PCR amplification results

**Fig. 3:**
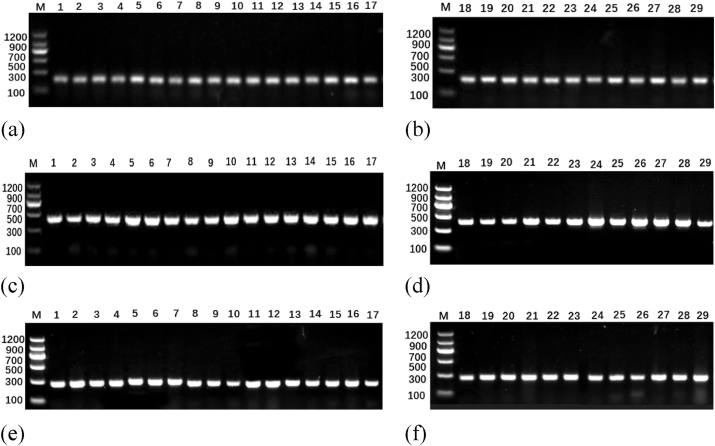
PCR products gel electrophoresis of single sample. Note: The electrophoresis results of samples 1-29 amplified by 12SA1 (a and b), 12SA3 (c and d) and 16S rRNA (e and f) primers; Lane M represents DNA Marker; Lane 1-29 represents pure samples.

Three sets of primers, 12SA1, 12SA2, and 12SA3, were optimized and screened. Electrophoresis results for these primer sets and the 16S rRNA primers showed that all primer sets, except 12SA2 (poor amplification performance, not shown in the figure), produced bright, single bands for each sample. This indicated that 12SA1 and 12SA3 primer sets were suitable for subsequent sequencing analysis. The consistent brightness of the PCR products confirmed that all samples met the amplification efficiency and sequencing requirements, with no non-specific amplification observed (Fig. 3).

Comparative sequencing analysis of the 12SA1 and 12SA3 primer sets revealed a higher success rate for 12SA3. Additionally, the PCR products from 12SA3 exhibited higher brightness compared to 12SA1 (Fig. 3). Based on these factors, the 12SA3 primer set was selected as the most suitable for further analyses.

#### 3.1.2 Comparison of species identification ability between different primers

All positive amplification products generated using the 12SA1, 12SA3, and 16S rRNA primers were directly sequenced. After proofreading and analysis, each sequence corresponded to species information with a percent identity exceeding 98%, satisfying the criteria for species identification (Table 4). However, due to the shorter amplified fragments produced by the 12SA1 and 16S rRNA primer sets, their species discrimination abilities were inferior to those of the 12SA3 primers (Table 4). Additionally, some sequences from the 12SA1 primer products exhibited bimodal patterns, complicating species identification.

**Table 4:**
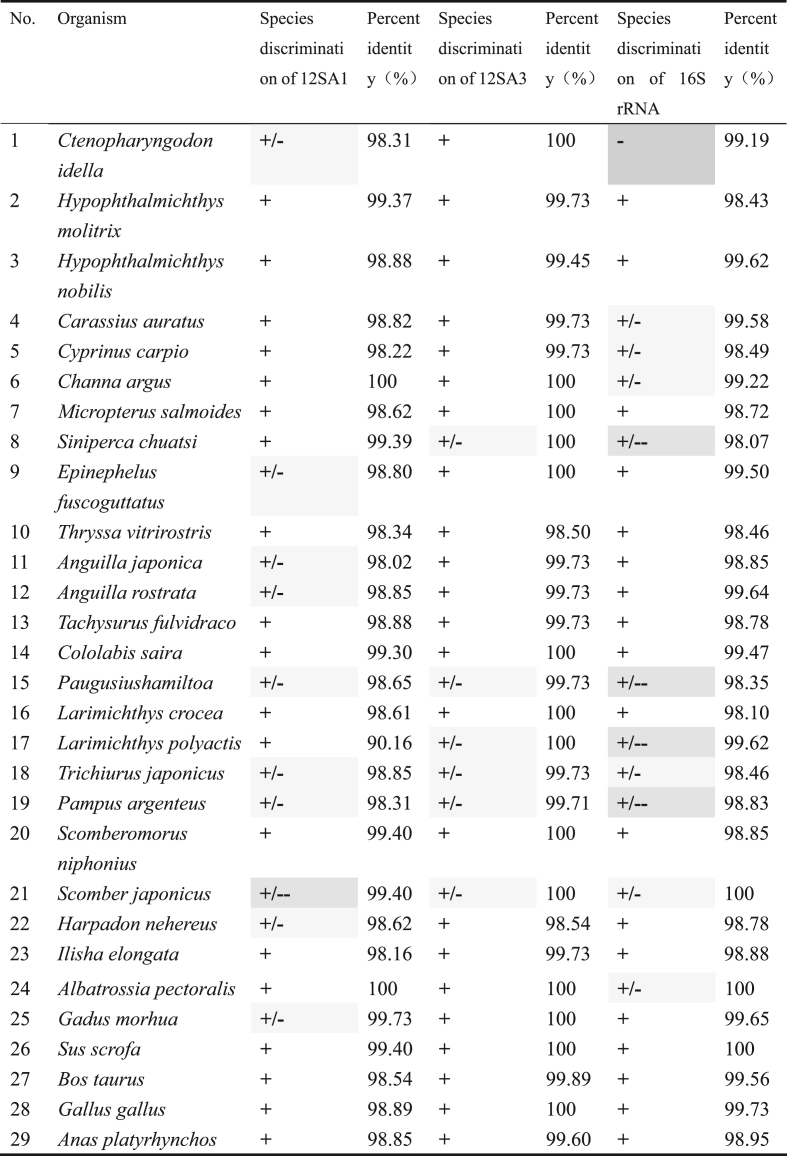
Identification results of 29 animal samples based on 12S rRNA gene and 16S rRNA gene.

Note: Comparison of the taxonomic resolution of different DNA barcodes for fish, livestock and poultry. A ‘+’ indicates that accurate identification of the species level; A ‘+/−’ indicates that poor species discrimination (two species of the same genus cannot be identified); A ‘+/−’ indicates that very poor species discrimination; A ‘-’ indicates that unidentifiable to species (The depth of grey represents the ability to discriminate between species, with darker colour representing poorer identification).

In contrast, the 12SA3 primer set demonstrated broad applicability and high amplification efficiency, enabling reliable differentiation of species at the genus or species level.

**Fig. 4:**
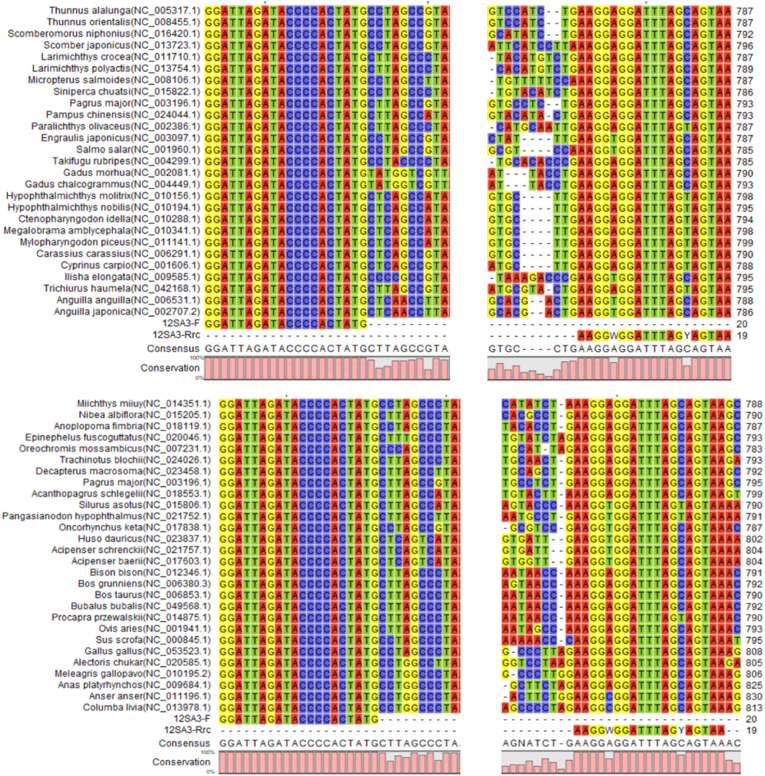
Alignment (CLC Genomics Workbench 8) of the barcoding region in 42 fishes and 13 livestock and poultry species (reference sequences taken from NCBI).

To further assess the suitability of the 12SA3 primers for identifying a broader range of raw materials used in fish balls, the 12S rRNA barcoding region of 42 fish and 13 livestock and poultry species (reference sequences from NCBI) was aligned using the CLC Genomics Workbench 8 (S and Angelika, 2023) (Fig. 4). The alignment results confirmed that the 12SA3 primer had wider applicability and higher amplification efficiency compared to the 16S rRNA primers (Sarri et al., 2014). These findings suggest that the 12SA3 primer not only enhances taxonomic resolution but also meets the read length requirements of the Illumina sequencing platform, making it more suitable for NGS analysis of fish ball samples.

### 3.2 Meta-barcoding analyses of manual fish balls

**Fig. 5:**
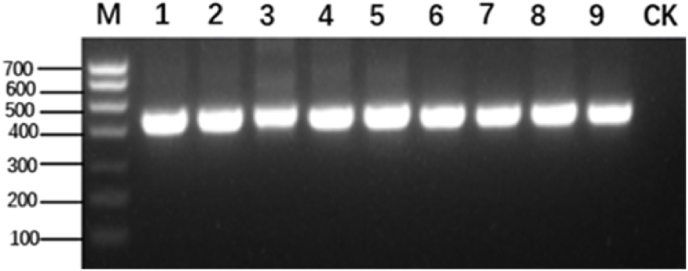
PCR products gel electrophoresis of manual samples. Note: Lane M represents DNA Marker; Lane 1-9 represents manual fish balls, CK represents negative control.

The manual fish ball DNA samples were amplified using the 12SA3 primer set. The resulting PCR products exhibited high specificity and quality, meeting the requirements for subsequent NGS (Fig. 5).

To assess the accuracy and sensitivity of the 12SA3 primer set in identifying mixed sample ingredients, Illumina PE250 sequencing was performed on samples N1–N9 of the manual fish balls. After quality control, 5,661,129 qualified reads were obtained, with lengths ranging from 302 bp to 350 bp.

**Fig. 6:**
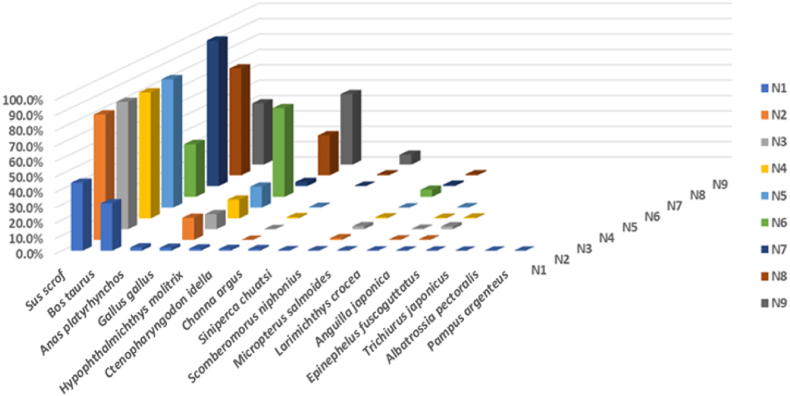
The results of next generation sequencing of N1-N9 of mixed DNA samples.

Comparison of the obtained sequences with the NCBI database confirmed that all mixed ingredients in each manual fish ball sample were accurately identified. Even samples containing *Larimichthys crocea*, *Ctenopharyngodon idella*, and *Scomberomorus niphonius*, designed with a minimum mixture of 1%, were successfully detected in samples N2, N3, and N5. Fig. 6 illustrates the relative abundances of each species in the mixed samples obtained from 9 manual fish ball samples through NGS.

In sample N1, 16 species were mixed in equal proportions; however, the sequencing results revealed that the relative abundances of each species did not match the initial proportions or satisfy a bisection relationship. Among these, *Sus scrofa* (44.148%) and *Bos taurus* (30.705%) showed significantly higher relative abundances than their actual weights, indicating that pigs and cows were dominant in the sequencing results for the mixed samples. Similarly, the relative abundances of species in the other 8 mixed samples also deviated from their initial proportions, further confirming that these results could not represent the species mixing ratios.

### 3.3 Meta-barcoding analyses of commercial fish balls

**Fig. 7:**
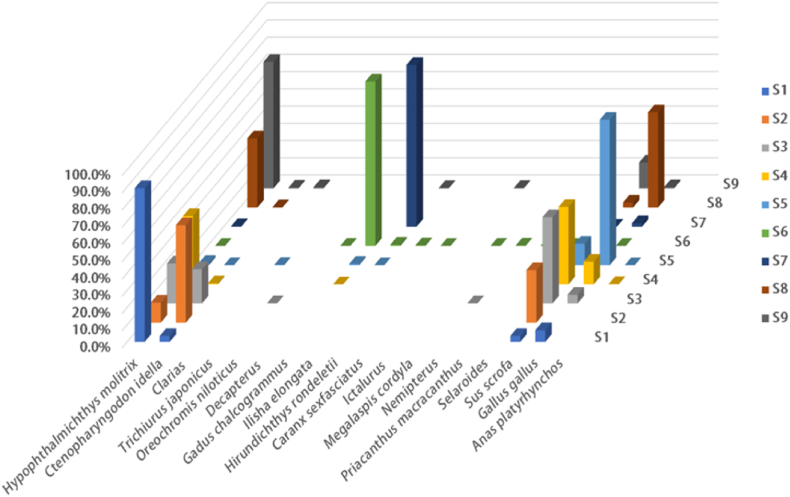
The results of next generation sequencing of commercial fish balls.

Nine commercial fish ball DNA samples were successfully amplified and sequenced. Based on the relative abundances of species (Fig. 7) and species identification results (Table 5), most fish balls were found to contain a mixture of two or more fish species, along with pig- and chicken-derived ingredients. However, The actual ingredients of two samples are, to a certain extent, inconsistent with their label information.

**Table 5:** Bioinformatics analysis results of commercial fish balls.

Fish balls ID	Label ingredients	Detected ingredients	Relative abundance (%)
S1	freshwater fish, pig, chicken	*Hypophthalmichthys molitrix*	89.2
		*Ctenopharyngodon idella*	0.14
		*Gallus gallus*	6.76
		*Sus scrofa*	3.42
		Others	0.48
S2	freshwater fish, pig, chicken	*Ctenopharyngodon idella*	56.58
		*Hypophthalmichthys molitrix*	11.63
		*Sus scrofa*	30.53
		*Gallus gallus*	0.51
		Others	0.75
S3	freshwater fish, pig, chicken	*Hypophthalmichthys molitrix*	23.22
		*Ctenopharyngodon idella*	19.95
		*Nemipterus*	0.13
		*Oreochromis niloticus*	0.14
		*Sus scrofa*	50.08
		*Gallus gallus*	5.06
		Others	1.42
S4	1.1 freshwater fish, pig, chicken, duck	*Hypophthalmichthys molitrix*	39.60
		*Ctenopharyngodon idella*	0.59
		*Gadus chalcogrammus*	0.04
		*Sus scrofa*	44.90
		*Gallus gallus*	13.18
		*Anas platyrhynchos*	0.19
		Others	1.50
S5	fish, pig, chicken	*Hypophthalmichthys molitrix*	1.35
		*Gadus chalcogrammus*	0.77
		*Trichiurus japonicus*	0.17
		*Ctenopharyngodon idella*	0.06
		*Ilisha elongat*	0.03
		*Gallus gallus*	84.39
		*Sus scrofa*	12.36
		*Anas platyrhynchos*	0.02
		Others	0.85
S6	Cod	*Gadus chalcogrammus*	95.47
		*Ilisha elongat*	0.74
		*Ctenopharyngodon idella*	0.59
		*Hirundichthys rondeletii*	0.33
		*Nemipterus*	0.17
		*Priacanthus macracanthus*	0.10
		*Caranx sexfasciatus*	0.05
		*Decapterus*	0.09
		*Megalaspis cordyla*	0.07
		*Selaroides*	0.05
		*Gallus gallus*	0.04
		Others	2.30
S7	marine fish, chicken	*Ilisha elongat*	93.92
		*Hypophthalmichthys molitrix*	0.39
		*Gallus gallus*	2.05
		*Sus scrofa*	0.03
		Others	3.61
S8	surimi, pig, egg white	*Hypophthalmichthys molitrix*	40.17
		*Ctenopharyngodon idella*	0.17
		*Sus scrofa*	2.91
		*Gallus gallus*	55.24
		Others	1.51
S9	surimi, pig, egg white	*Hypophthalmichthys molitrix*	73.39
		*Clarias*	0.67
		*Ctenopharyngodon idella*	0.42
		*Ictalurus*	0.22
		*Ilisha elongata*	0.18
		*Sus scrofa*	14.99
		*Gallus gallus*	0.70
		Others	9.43

For sample S5, the analysis revealed a complex composition, with the main ingredient being chicken-derived (84.39%) and mixed with various marine and freshwater fish. In sample S6, the primary raw material was *Gadus chalcogrammus* (Alaska pollock, 95.47%), along with *Ilisha elongata* (Bennett, 0.74%), *Ctenopharyngodon idella* (grass carp, 0.59%), and trace amounts of other marine fish.

The analysis of commercial fish ball samples demonstrated that refined DNA meta-barcoding offers wide applicability and high practicality, making it a reliable tool for species identification in mixed products like fish balls.

## 4 Discussions

In recent years, adulteration in processed seafood has persisted despite regulatory prohibitions, impacting product quality, aquaculture, production, sales, and consumer rights. DNA meta-barcoding, combined with Next-Generation Sequencing (NGS), has emerged as a powerful alternative to traditional methods such as morphological, physical, and chemical identification, as well as Sanger sequencing, for processed seafood. It is widely applied in identifying animal-derived ingredient adulteration in complex foods (Mottola et al., 2022; Piredda et al., 2022). For example, Giusti et al., (2017) used 16S rRNA meta-barcoding to analyze commercial surimi samples and found that 37.5% of the products were mislabeled, detecting multiple unidentified fish and cephalopod species. Similarly, Carvalho et al., (2017) analyzed 22 processed cod products using COI and CytB gene barcoding and discovered that 31% contained a mixture of two or more species, with a mislabeling rate of 41%. These findings demonstrate the high applicability of DNA meta-barcoding and NGS in detecting surimi product adulteration. But, on the other hand, it is difficult to identify the species of a mixture solely based on a single DNA barcode, and it requires the use of DNA meta-barcoding with universal primers or multiple detections (Zhang et al., 2024).

In this study, 12S rRNA was selected and a meta-barcoding assay was developed for identifying multiple species in fish balls. Suitable primers were designed to identify approximately 30 commonly used animal-derived ingredients, including 26 fish species, 2 mammals, and 2 poultry species. A short DNA barcoding region with high versatility, sensitivity, and specificity was screened across the 30 species mentioned. An ideal meta-barcoding primer set should have short amplification lengths, broad taxonomic coverage, high resolution, specificity, and unbiased amplification (Shu et al., 2021). This 12S A3 primer set precisely meets this ideal criterion. It was reported that 12S rRNA primers could detect more species than 16S rRNA or COI primers (Girish et al., 2022; Jean, 2021; Zhang et al., 2020), which indicated that 12S rRNA gene was more suitable for multiple species identification. In this study, 12S rRNA meta-barcoding detection system has been proven to possess the above advantages, which is exactly why 12S rRNA was selected as the target in this study.

The 12S rRNA gene had been used for meta-barcoding of fish in environmental DNA (eDNA) studies, and has shown promise in detecting fish diversity using primers like AcMDB07 and MiFish-U (Noh et al., 2021). However, further analysis revealed mismatched bases in these primers when targeting avian, fish, and livestock DNA (Shu et al., 2021). These mismatches could result in false negatives in samples with non-target species and unknown ingredients. To address this limitation, the optimal universal primer set, 12SA3, was designed for identifying common ingredients in fish ball products. Furthermore, comparative experimental results indicated that the 12SA3 primer set possessed high amplification efficiency, taxonomic resolution, and superior identification ability compared to 16S rRNA primers. NGS results indicated that the primer set could accurately identify all mixed ingredients in manual fish balls, including ingredients present at a minimum of 1%, highlighting its sensitivity and practical application. However, we found that the relative abundance of different species detected by NGS did not correspond linearly to their initial proportions in the samples, consistent with findings by Wang et al., (2021). Quantitative detection of each component in mixed samples was not feasible due to differences in genomic DNA extraction efficiency, primer amplification efficiency, and variations in mitochondrial content, fat, and protein among species (Ballin et al., 2009).

Additionally, trace false-positive results were observed across all 9 samples, likely due to PCR product contamination by aerosols during sequencing. These false positives did not affect the overall results, as a 0.02% sequencing read threshold was set to exclude them. Excluding the above factors, the 12S rRNA meta-barcoding primer (12SA3) designed in this study is highly effective for qualitative detection of fish-, livestock-, and poultry-derived ingredients in fish balls. While it accurately identifies mixed species, it cannot infer their proportions based on relative abundance, making it suitable for qualitative but not quantitative analysis.

The product labels of commercial fish balls collected in this study generally lacked clear species information and were often labeled with vague terms such as “marine fish,” “freshwater fish,” or “surimi”. The ingredient analysis of 9 commercial fish balls using 12S rRNA revealed that each product contained two or more fish species. Manufacturers likely used seawater fish surimi mixed with freshwater fish surimi to reduce costs and improve the gel properties of fish balls (Song and Lin, 2020). For example, grass carp was detected in sample S6 and silver carp in sample S7. Additionally, some illegal traders directly used untreated fish intestinal for fish ball production. The complex ingredients in intestinal contents could contribute to low sequencing abundance and false-positive results (Ueki et al., 2016).

Furthermore, the high sensitivity of NGS detected non-labeled contaminating ingredients, likely originating from residual raw materials from other products on the fish ball production line, further contributing to false-positive results.

In summary, fish ball production is complex and susceptible to adulteration. Regulatory authorities must enhance their capacity to identify and detect surimi products like fish balls, ensuring effective supervision and control of adulterated ingredients and impurities. Meanwhile, referring to quantitative detection methods for other food ingredients is also an important direction to improve the adulteration detection capability of fish balls, so as to further ensure the healthy development of the fish ball industry (Liang et al., 2022; Caballero-Agosto et al., 2026).

## 5 Conclusion

This study redesigned and optimized a set of universal primers based on the 12S rRNA gene for detecting fish-, livestock-, and poultry-derived ingredients in commercial fish balls. Sequencing and identification of single and mixed samples demonstrated that the selected primers have higher taxonomic resolution, improved universality and accuracy, and sensitivity sufficient to detect species at a rate as low as 1%. Analysis of commercial fish ball products revealed significant discrepancies in two samples compared to their labels, confirming the presence of adulteration and highlighting the necessity of testing.

In conclusion, this study provides a set of meta-barcoding primers suitable for qualitative identification of mixed ingredients in fish products, serving as an important complement to existing DNA meta-barcoding techniques.

## Ethical approval

No animal or human subject was used in the work related to this manuscript.

## Author contribution

Feng Guan: Writing – review & editing, Writing – original draft. Qingqing Hu: Writing –original draft, sequences analysis, Visualization, Validation, Methodology, Investigation, Data curation. Siyu Yang: Methodology, Investigation, Data curation. Shimeng Dai: Data analysis. Jian Ge: Experiment design, original draft, translation. Xingjuan Hu: Funding acquisition, Conceptualization. All authors have read and approved the published version of the manuscript.

## Declaration of competing interest

All the authors declare no competing interests.
